# Pathway mapping and development of disease-specific biomarkers: protein-based network biomarkers

**DOI:** 10.1111/jcmm.12447

**Published:** 2015-01-05

**Authors:** Hao Chen, Zhitu Zhu, Yichun Zhu, Jian Wang, Yunqing Mei, Yunfeng Cheng

**Affiliations:** a Department of Cardiothoracic Surgery, Tongji Hospital, Tongji UniversityShanghai, China; b Department of Tumor Medicine, Jingzhou Hospital, Liaoning Medical UniversityJingzhou, China; c Department of Respiratory Medicine, Fudan UniversityShanghai, China; d Department of Hematology, Fudan UniversityShanghai, China; e Biomedical Research Center, Zhongshan Hospital, Fudan UniversityShanghai, China; f Center for Clinical Bioinformatics, Fudan UniversityShanghai, China

**Keywords:** network biomarkers, dynamic network biomarkers, protein–protein interactions, disease, diagnostics

## Abstract

It is known that a disease is rarely a consequence of an abnormality of a single gene, but reflects the interactions of various processes in a complex network. Annotated molecular networks offer new opportunities to understand diseases within a systems biology framework and provide an excellent substrate for network-based identification of biomarkers. The network biomarkers and dynamic network biomarkers (DNBs) represent new types of biomarkers with protein–protein or gene–gene interactions that can be monitored and evaluated at different stages and time-points during development of disease. Clinical bioinformatics as a new way to combine clinical measurements and signs with human tissue-generated bioinformatics is crucial to translate biomarkers into clinical application, validate the disease specificity, and understand the role of biomarkers in clinical settings. In this article, the recent advances and developments on network biomarkers and DNBs are comprehensively reviewed. How network biomarkers help a better understanding of molecular mechanism of diseases, the advantages and constraints of network biomarkers for clinical application, clinical bioinformatics as a bridge to the development of diseases-specific, stage-specific, severity-specific and therapy predictive biomarkers, and the potentials of network biomarkers are also discussed.

- Introduction
- The need and significance of protein-based network biomarkers
- The development of protein-based network biomarkers
  –Human protein–protein interaction network
  –Methodologies for integrating and identifying network biomarkers
  –Network biomarker studies in humans
  –Better understanding of molecular mechanism of diseases
- Correlation between network biomarkers and clinical informatics
- The advantages and constraints for clinical application
- Prospective and conclusions

## Introduction

**Fig 1:**
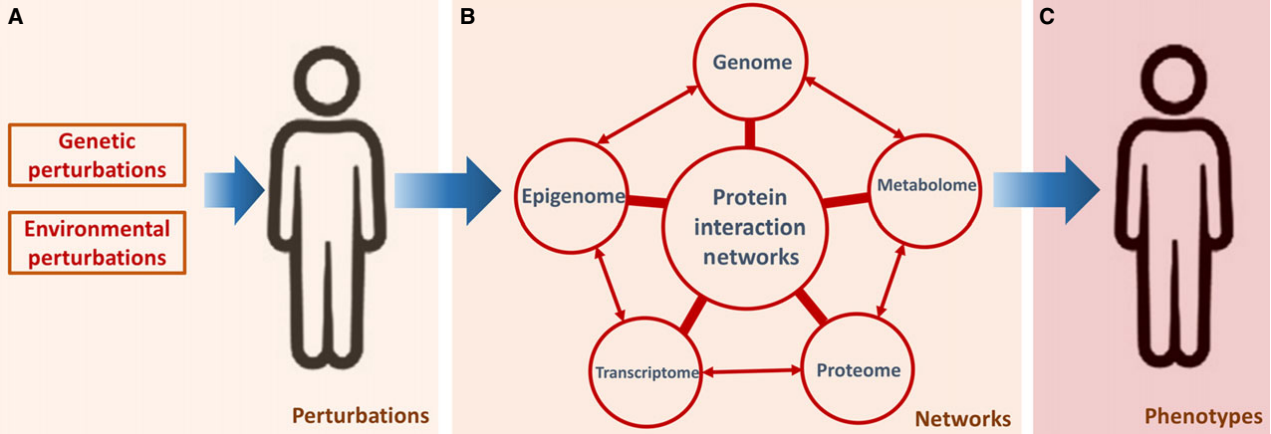
Network-based human disease model. In response to the dynamically varying intrinsic (genetic) and extrinsic (environmental) perturbations (A), protein interaction networks (B) that interactively link genome, epigenome, transcriptome, proteome and metabolome are of central importance to modulate cell behaviour. The interplay of these interconnected cellular signalling networks can converge towards disease states and ultimately can initiate and drive complex diseases (C).

The disease consists of multiple dysfunctional proteins, cells, organs and systems of the body within the complexity and molecular mechanisms by which diseases occur remain unclear, although biotechnologies and knowledge on diseases have been improved tremendously. Given the functional interdependencies between the molecular components, a disease is rarely a consequence of an abnormality of a gene, protein or cell, but reflects the interactions of genes, proteins or cells in a complex network 1. Protein–protein interactions (PPIs) are important in the interaction, communication and functional process in a living cell, between cells, or between organs. PPI networks are of central importance to modulate cell behaviour by interactively link genome, epigenome, transcriptome, proteome and metabolome. In response to the dynamically varying intrinsic (genetic) and extrinsic (environmental) perturbations, the interplay of these interconnected cellular signalling networks can converge towards disease states and ultimately can initiate and drive complex diseases (Fig.1). Annotated data sets of PPIs offer new opportunities to understand diseases within a systems biology-based framework and provide a useful substrate for network-based identification and validation of multiple interacting markers 2,3. The PPIs are curated from the literature into databases 4, to improve the understanding of diseases and provide the basis for new therapeutic approaches.

Biomarkers are typically thought of as individual genes, proteins and metabolites (molecular biomarkers). However, with the recent innovation and progress of new biotechnologies, a new type of biomarkers, protein-based network biomarkers 5,6, composes of a panel of proteins and their interactions or interactions with DNA, RNA or other molecules was initiated and investigated with the integration of knowledge on protein annotations, interactions and signalling pathways. But both the molecular biomarkers and the protein-based network biomarkers have limitations because of their static nature. To increase the ability to make early diagnosis, identify disease-specific biomarkers and therapeutic targets, and predict patient outcome, dynamic network biomarkers (DNBs) were created, monitored and evaluated at different stages and time-points of the disease, based on non-linear dynamical theory and complex network theory. DNBs differ from molecular biomarkers and network biomarkers to describe and identify disease progress situations and interactions rather than the static nature and approach. Thus, DNBs can demonstrate the expression density of genes or proteins and their time-dependent interactions to draw a three-dimensional imaging of altered proteins, interactions or regulations in the network and to discover and develop disease-specific biomarkers to predict and foresee pre-disease situations, monitor and regulate therapeutics in clinic, and indicate and guide of patient prognosis and life quality 7,8.

Furthermore, the human biomarker discovery mandates the association of biological measurements with clinical information, ideally both statistically and mechanistically. Unlike biological data, clinical data, such as patient complaints, history, therapies, clinical symptoms and signs, physical examinations, biochemical analyses, imaging profiles, pathologies and other measurements, are descriptive and far less structured. The lack of integrative results in the loss or neglecting of valuable information, necessitates novel strategies to successfully combine these large collections of heterogeneous data sets, and identifies new disease-specific biomarkers. To address this issue, clinical bioinformatics was proposed as a new emerging science to combine clinical phenotypes with human tissue-generated bioinformatics and define relationships between collectively directs global function with clinical measurements 9,10. Comparing dynamic alterations of network biomarkers with clinical informatics may allow discovering disease-specific, stage-specific, severity-specific and therapy-sensitive biomarkers.

The present review aims to introduce the need and significance of protein-based network biomarkers, highlight the development of network biomarkers of human diseases, and discuss the clinical relevance and correlation between DNBs and clinical informatics. We will explore how network biomarkers help a better understanding of molecular mechanism of diseases, the advantages and constraints of network biomarkers for clinical application, and the potential values of network biomarkers in the future.

## The need and significance of protein-based network biomarkers

Biomarkers play an important role in the diagnosis of diseases, and in assessing prognosis and directing treatment of the diseases. Its value is correlated with the disease-associated specificity, sensitivity, traceability, stability, repeatability and reliability. Conventional molecular biomarkers consist of single or a group of several biological molecules such as genes, RNAs, proteins and metabolites that can be measured to distinguish disease from health. Advances in high-throughput technologies such as genomics and proteomics make it possible to measure thousands of different variables in pathobiological conditions simultaneously, providing comprehensive and substantial information of a disease state. An increasing number of biomarkers identified through analysis of expression profiles have been seen. Unfortunately, this type of biomarker lists that obtained from omics data for similarly diagnosed patients by different research groups differ widely and share few in common 11. As biomarkers are required to be reproducible to causally link to the disease phenotype to discover potential targets for diagnosis and therapy, this lack of agreement imposed doubts about the reliability and robustness of the reported biomarker lists.

The small overlap of biomarkers for similar phenotypes may have various technique reasons, such as platforms differences, samples differences, protocols differences, and statistical reasons, leading to unstable selection of the biomarkers. Besides, identification of a phenotype-associated pathway solely on the basis of differentially expressed molecules is frequently difficult because of the high interdependency of the omics data. But more importantly, these biomarkers are not identified from the systems perspective. With the rapid growing knowledge of the cellular molecular network in diseases, most of the diseases are not considered to be caused by a single effector gene product but the interrelated malfunction of genes and proteins 1,12–14. This system-level understanding of the diseases has brought about novel strategies of biomarker discovery that integrate systemic information of the molecular networks (*i.e*. PPI networks, RNA networks, metabolic networks and regulatory networks) to contextualize the differential expression patterns observed in a phenotype. The system-based ‘network biomarkers’ 5,6 was proposed to consider not only differentially expressed molecules but also the molecules association network structure that even allows an accurate identification of biomarkers with low discriminative potentials provided such molecules were associated with other significant molecules. The past few years have witnessed systematic efforts to integrate gene network knowledge in the gene expression analysis 15–19. However, a clearly limitation of gene network analysis is that genes are not the proper end-point context of a phenotype. Moreover, the majority of human genes have not yet been assigned to a definitive pathway. Proteomic profiling provides information at the post-translational level, therefore can be used to bridge the genotype-phenotype gap, to help providing a global picture of cellular mechanisms. With the recent significant improvements of mapping human protein networks, network approaches have been studied in proteomic researches to understand disease-related pathobiological processes and to identify candidate disease biomarkers 6,7,20–24.

Studies have also integrated multiple data types to generate more accurate molecular networks of diseases 5,25–46 and revealed the dynamic modular structure of the protein interaction networks 47–49. Integration of condition-specific co-expression information can provide clues to the dynamic features of these networks, when PPI data constitute static network maps – such knowledge-integrated interaction is relatively defined and fixed. The ‘DNBs’ is an innovative concept to integrate network biomarkers and dynamic biomarkers by monitoring and evaluating the alterations of network biomarkers at different stages and time-points. Unlike static molecular biomarkers and network biomarkers with consistent values, DNB is a group of molecules, which are highly fluctuating but strongly correlated without consistent values during disease progression 7. DNB is a powerful way to detect the bifurcation of gene or protein interactions to unravel the dynamic aspects of cellular networks and answer when, where and why proteins interact, and to indicate the early change in biomarkers and to predict the occurrence of diseases 50,51.

Chen *et al*. 7 proposed to consider a disease progress as three stages, including the normal stage, the pre-disease stage and the disease stage. The normal stage is a relatively healthy state, as well as the chronic inflammation period or the period that the disease is under control. The pre-disease stage 52–55 is a state just before the presence of disease symptoms, defined as the limit of the normal state immediately before the critical point is reached. This stage is usually reversible to the normal state if appropriately treated, or becomes irreversible if the system passes over the critical point to the disease stage. Therefore, detection of the pre-disease stage is crucial to achieve early diagnose and treatment. Rather as a wide, general definition been discussed before, the authors developed DNB in particular as an early warning indicator of pre-disease state and to fulfil three criteria 7: firstly, DNB is an observable subnetwork of the system, composed of a group of molecules that are dynamically correlated when the system is in a pre-disease state; secondly, DNB is an isolated subnetwork or functional module, behaves independent of other non-DNB molecules that are in the same system or network; and thirdly the expressions of DNB molecules increasingly fluctuate as the system is approaching the critical point. Based on these conditions, a composite index *I* was constructed to computationally identify DNB from high-throughput omic data:

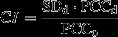
where PCC_d_ is the numerical measurement of the average Pearson's correlation coefficient among the molecules of DNB in absolute value; PCC_o_ is the numerical measurement of the average Pearson's correlation coefficient of the molecules of DNB with the other molecules in absolute value; SD_d_ is the numerical measurement of the average standard deviation of the molecules of DNB. The composite index *I* was shown to be effective to provide reliable and significant early warning signal, despite the stochastically fluctuation in the expression of each molecule, in complex diseases such as acute lung injury, liver cancer and lymphoma 7.Furthermore, DNB was demonstrated as the leading/driving network causally related to disease initiation and progression 8.

**Fig 2:**
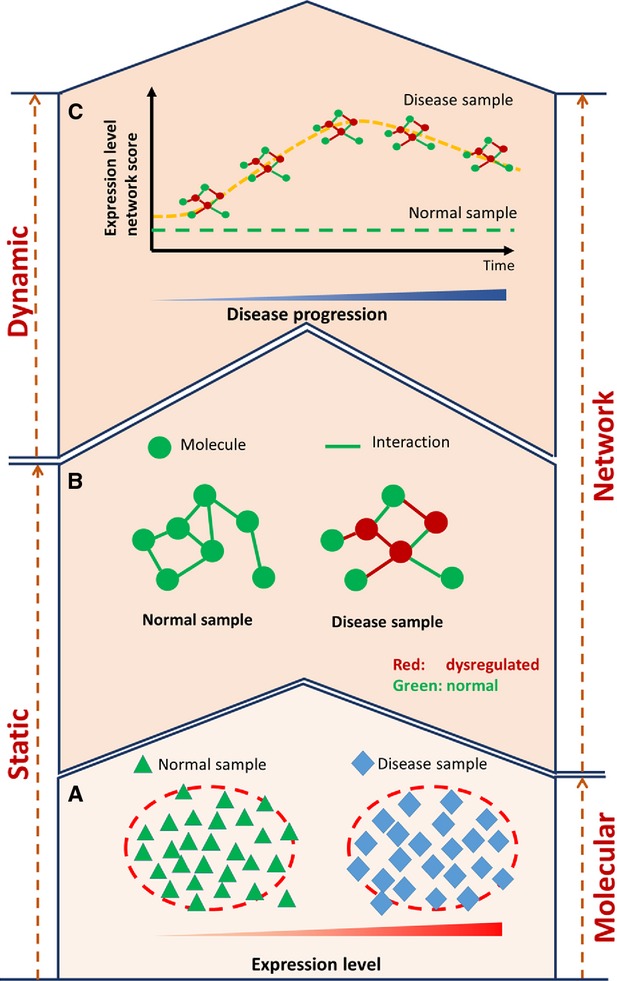
The evolution of biomarkers concepts. (A) Traditional molecule biomarkers, known as single or a group of several genes and proteins that are static indicators on the disease state; (B) The recent developed network biomarkers, with the integration of knowledge on protein annotations, interactions, and signalling pathways, are static measurements on the disease state; (C) The newly developed dynamic network biomarkers, providing dynamical measurements on the disease state within a systems biology framework.

Figure2 summarizes the evolution of these three types of biomarker concepts, namely, molecular biomarkers that provide static, one-dimensional information (Fig.2A), network biomarkers that provide static, two-dimensional profiles (Fig.2B), and DNBs that provide dynamic, three-dimensional images of biomarker–biomarker interactions (Fig.2C).

## The development of protein-based network biomarkers

**Fig 3:**
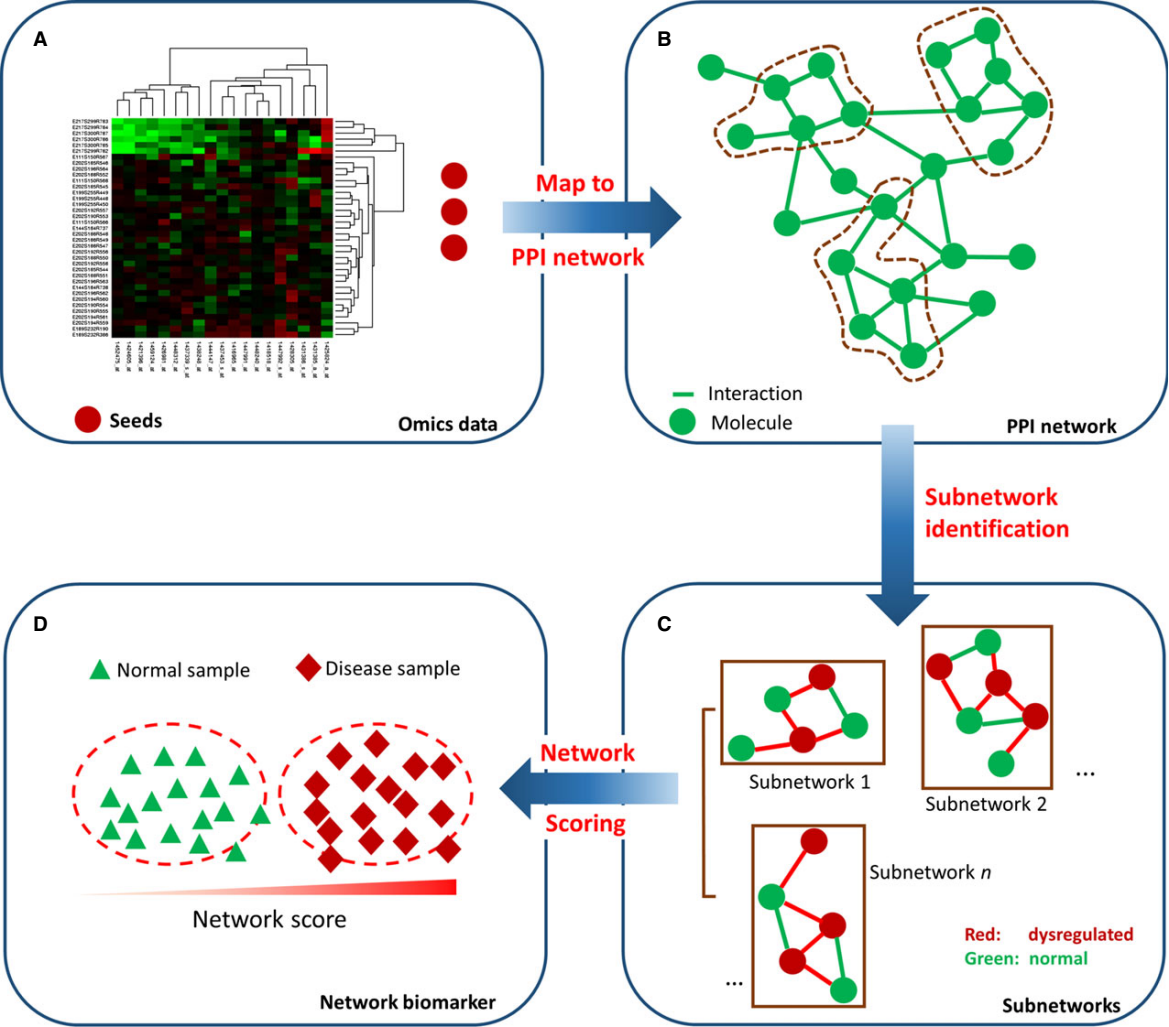
Systematic approach of network biomarker discovery. Chart schematically illustrates the critical stages of network biomarker discovery: (A) The global expression profiles (genomics, proteomics, literatures, *etc*.) of a disease phenotype are obtained as ‘seeds’ of the disease module; (B) Such seeds is integrated into the constructed protein network (Y2H, AP-MS), literature-curated pathways, or computational predicted networks that contain systematic pathobiological events of phenotypes; (C) Through quantitative systematic approaches, phenotype-associated subnetworks and/or pathways are then scored, ranked and identified; and (D) such disease modules or network biomarkers can distinguish a disease phenotype from a normal phenotype more accurately than traditional molecule biomarkers. Y2H: high-throughput yeast two-hybrid; AP-MS: affinity purification mass spectrometry.

Protein–protein interactions play major role in a living system, provide information at the post-translational level, and bridge the genotype–phenotype gap. Annotated data sets of PPIs provide key substrate for network-based identification of biomarkers. The systematic approach of protein-based network biomarker discovery typically involves three pivotal processes: (*i*) obtaining global expression profiles of a disease phenotype; (*ii*) integrating such information into protein network frameworks or literature-curated pathways that contain key pathobiological events of phenotypes and (*iii*) interpreting, identifying and validating phenotype-associated candidate key network molecules or modules (Fig.3). This section mainly introduces some foundations of protein-based network biomarker discovery and demonstrates several representative studies in human diseases and how such network biomarkers shed light onto the molecular mechanism of diseases.

### Human protein–protein interaction network

Human PPI network mapping is a crucial component of systematic approach for protein-based network biomarker discovery. Extensive efforts have been seen recently to increase the coverage of human PPI maps by high-throughput yeast two-hybrid (Y2H) interaction mating 2,3,56–58, affinity purification mass spectrometry (AP-MS) 59, literature curation of published experiments 60–70 or computational integrating approaches 71–76. Some of the major public PPI databases are summarized in Table1. The global efforts to map protein interactions with curated interactions from the literature also has resulted in the formation of the International Molecular Exchange (IMEx) consortium (http://www.imexconsortium.org/) which can facilitate literature curation standards, data exchange and comparison. However, considering the full human protein interaction network has been estimated to be between 154,000 and 369,000 77 or approximately 650,000 78, current human PPI maps are still incomplete, noisy and prone to biases 74,79, caution should be exercised when using them to research diseases. Given the magnitude of this challenge, a number of strategies have been proposed to maximize the efficiency and correct biases for PPI network mapping 56–58,80.

**Table 1:** Information of human protein–protein interaction network databases

Database	Method	Proteins number	Interactions number	Ref.	Website
CCSB	Y2H	1549	2754	2	http://interactome.dfci.harvard.edu/
MDC	Y2H	1705	3186	3	http://www.mdc-berlin.de/neuroprot/database.htm
BIND	Literature	6089	14955	60	http://bind.ca
DIP	Literature	3877	6103	62	http://dip.doe-mbi.ucla.edu/dip
MINT	Literature	8762	26830	63	http://mint.bio.uniroma2.it/mint
HomoMINT	Prediction & Literature	8634	323595	70	http://mint.bio.uniroma2.it/HomoMINT
InteAct	Literature	60932	197974	64	http://www.ebi.ac.uk/intact/
BioGRID	Literature	18208	220390	65	http://thebiogrid.org/
HPRD	Literature	30047	41327	66	http://hprd.org/
Reactome	Literature	7085	6744	68	http://www.reactome.org/
UniHI	Database integration	36023*	374833*	74	http://www.unihi.org/
HAPPI	Database integration	70829	601757	75	http://bio.informatics.iupui.edu/HAPPI

The table displays the number of proteins and the number of interactions derived from each database. The column termed Methods shows the general approaches how PPIs were compiled in the different resources. In addition, the literature reference for the resource and the websites of the databases are given.

CCSB: Center for Cancer Systems Biology; MDC: Max Delbrück Center; BIND: Biomolecular Interaction Network Database; DIP: Database of Interacting Proteins; MINT: Molecular INTeraction database; HomoMINT: inferred human network of MINT; InteAct: the protein Interaction database; BioGRID: Biological General Repository for Interaction Datasets; HPRD: Human Protein Reference Database; Reactome: a curated pathway database; UniHI: Unified Human Interactome; HAPPI: Human Annotated and Predicted Protein Interaction.

* All species.

### Methodologies for integrating and identifying network biomarkers

A disease can be linked to a well-defined neighbourhood of PPI network, which refers to as ‘disease module’, representing a panel of network components responsible for cellular function and disruption of which results in a specific disease phenotype 1. Disease-related profiles can be integrated within a network framework by a number of technologies and algorithms 81,82. Computational programs were developed to integrate selected genes or proteins into the knowledge-based networks *via* the combination of genomics, proteomics and bioinformatics, such as GRNInfer 83, MDCinfer 84, TRNInfer 85, Samo 86, MNAligner 87, PTG 88, PRNA 89, NOA 90, differential dependency network (DDN) 91, WGCNA 92, SurvNet 93 or DiME 94, each of them has its own advantages and strength on basis of scientific needs and investigative goals, as summarized in Table2.

**Table 2:** Examples of software packages for mapping phenotype-related subnetworks

Name	Full name	Website	Description	Ref.
GRNInfer	Gene Regulatory Network Inference Tool	http://digbio.missouri.edu/grninfer/	A gene regulatory network inference tool from multiple microarray data sets	83
MDCinfer	Inferring protein–protein interactions based on multi-domain Co-operation	http://intelligent.eic.osaka-sandai.ac.jp/chenen/MDCinfer.htm	PPI prediction tool based on multiple domain co-operation analysis	85
TRNInfer	Inferring transcriptional regulatory networks from high-throughput data	http://intelligent.eic.osaka-sandai.ac.jp/chenen/TRNinfer.htm	Infer direct relationships between transcription factors and target genes	85
Samo	Protein Structure Alignment tool based on Multiple Objective optimization	http://doc.aporc.org/wiki/Samo	A protein structure alignment tool based on multiple objective optimization	86
MNAligner	Molecular Network Aligner	http://doc.aporc.org/wiki/MNAligner	Alignment of molecular networks by quadratic programming	87
PTG	Parsimonious Tree-Grow method for haplotype inference	http://doc.aporc.org/wiki/PTG	Parsimonious tree-grow method for haplotype inference	88
PRNA	Protein–RNA Binding-Site Prediction	http://doc.aporc.org/wiki/PRNA	Prediction of protein–RNA binding sites by a random forest method with combined features	89
NOA	Network Ontology Analysis	http://www.aporc.org/noa/	Collection of gene ontology tools aiming to analyse functions of gene network instead of gene list	90
DDN	Differential dependency network analysis	http://www.cbil.ece.vt.edu/software.htm	Detect statistically significant topological changes in the transcriptional networks between two biological conditions	91
WGCNA	Weighted correlation network analysis	http://www.genetics.ucla.edu/labs/horvath/CoexpressionNetwork/Rpackages/WGCNA	A comprehensive collection of R functions for performing various aspects of weighted correlation network analysis patterns among genes across microarray samples	92
SurvNet	N/A	http://bioinformatics.mdanderson.org/SurvNet	A bioinformatics web app for identifying network-based biomarkers that most correlate with patient survival data	93
DiME	Disease Module Extraction	www.cs.bham.ac.uk/∽szh/DiME	A novel algorithm based on the Community Extraction criterion, to extract topological core modules from biological networks as putative disease modules	94

The table displays the abbreviated name and full name of the computational programs with respective description and website. In addition, the literature reference for the resource is given.

Methodologies for expression data integration could be categorized as ‘univariate’ or ‘multivariate’ on basis of statistics 95, to interpret the dysregulation (differential expression) at the system level. Defining the subnetworks dysregulation as the aggregate significance of the dysregulation of each gene, the univariate approaches measure the dysregulation of subnetworks by combining the results of differential expression of each gene that are assessed separately 47,49,96–99. However, multivariate studies consider the dysregulation of the subnetworks as the mutual information between phenotype and subnetworks activity, and access the coordination of multiple gene differential expressions in discriminating normal and disease samples 99. The coordinate dysregulation was shown to be effective integrating protein and mRNA expression data for identification of important subnetworks in colorectal cancer 30,33. The coordination of subnetwork dysregulation could be captured by ‘additive’ or ‘combinatorial’ formulations. The additive dysregulation formulates the coordination between genes through the additive representation of their expression profiles and utilizes fast heuristics to identify dysregulated subnetwork; while the combinatorial formulation assesses the combining degrees of gene expressions in the subnetwork that can discriminate control and phenotype samples. The combinatorial approach was shown to be powerful in predicting liver metastasis in human colorectal cancer 100. As the coordinate dysregulation that is not explained by smaller parts of the subnetwork, the synergistic dysregulation was formulated to delineate the complementarity of genes in the subnetwork 101. Synergy corrects for the coordinate dysregulation of the subsets of the subnetwork, capturing the pattern of dysregulation that emerges only when all genes in the subnetwork are considered. Although identification of multiple genes with synergistic dysregulation is intractable, this methodology provides important insight through pair-wise assessment of synergy, which jointly analyses two sets of expression data, one in the presence and one in the absence of a disease, identifying gene pairs whose correlation with disease is because of co-operative, rather than independent, contributions of genes 102. Besides coordinate dysregulation, differential co-expression approaches are also shown to be effective in finding co-expressed genes in the disease samples, rather than controls, and vice versa 103,104.

Briefly, disease-related molecules identified from omic profiling studies or other sources, which serve as the ‘seeds’ of the disease module, are placed on their corresponding proteins in the properly constructed PPI network, and by exploiting both the functional and topological modularity of the network through quantitative systematic approaches, subnetworks and/or pathways with the disease-related components could then be identified as disease module, and as potential network biomarker. Numerous computational methods and algorithms have been proposed for network biomarker identification. For instance, the DDN analysis 91,105, which detects topological changes in biological networks by comparing the topological differences between networks, is a straightforward way in distinguishing disease samples when the topology of disease networks is significantly different from the topology of normal samples. However, the network structure learning is inconsistent because of the limitation of the data samples, making this approach not convenient in real applications. The active subnetwork identification approach 5,106 identifies disease module as active subnetworks that show significant changes in particular conditions by using existing PPI networks. While this approach can identify disease-related subnetworks that are not differentially expressed, it is limited by the availability of the PPI networks. The disease-specific pathway identification method 37,73,107 is another systemic approach to extract disease-specific subnetworks or pathways by using regression models or scoring modules. This approach is effective in identifying network biomarkers based on the integration of PPIs and pathway knowledge. However, it is limited by the exhaustive search procedure. Besides, caution should be excised that the regression model-based method is not suitable for small sample cases, in which the parameters are biased. The information flow modelling approach 108,109 identifies dysfunctional modules in complex disease by modelling the information flow from source disease genes to targets of differentially expressed genes *via* a context-specific PPI network. This approach is effective in characterizing the functional dependency or crosstalk between pathways provided differentially expressions are detected. Unlike the conventional clustering approaches based on differential expressions, classification of differential interactions 110,111 investigates the differential interactions between disease and normal samples and network rewiring between molecules related to pathogenesis. Applied to gastric cancer, this method demonstrated that the differential interactions are effective on identifying dysfunctional modules from the molecular interaction network and can be applied as network biomarkers 111. However, this approach is time consuming because of the large-scale interaction networks. The supporting vector machine (SVM) approach 35,112,113 identifies a comprehensive key interaction map and integrating different types of interaction information of heterogeneous data sources within the SVM scheme. By using various biological knowledge and data sources such as gene co-expression, regulatory networks, evolutionary relationship and functional similarity, the effectiveness and efficiency are significantly improved. The major shortcoming of SVM is its high computational cost for real applications.

### Network biomarker studies in humans

Variants of aforementioned approaches have been applied to a wide range of disease phenotypes for identifying protein-based network biomarkers, such as breast cancer 5,35,36,49, colorectal cancer 30,33,100, prostate cancer 97,114, gastric cancer 115, lung cancer 37, ovarian cancer 116, acute myeloid leukaemia 39, glioma 117, ageing 23,98, Alzheimer's disease 38, inherited ataxias 118, cardiovascular diseases [6,32,34,109,119,120], chronic obstructive pulmonary disease 22, diabetes 43, asthma 121, osteoarthritis 42,122, multiple sclerosis 29, primary immunodeficiency 31, systemic inflammation 25, or acute aortic dissection 123, and demonstrated promising results. For example, Jin *et al*. 6developed a cardiovascular-related network based on protein information databases, and discovered network biomarkers of major adverse cardiac events (MACE) in the MS data on basis of protein knowledge. Candidate network biomarkers could classify patients with MACE more accurately than current single ones without network information. Similarly, Lim *et al*. 118 developed a PPI network of 54 proteins involved in 23 inherited ataxias, and expanded the network by incorporating literature-curated and evolutionarily conserved interactions. This phenotype-based PPI network reveals several previously unsuspected ataxia-causing proteins interactions and provides candidate genes for target-based therapies.

Integrating multiple types of data sources could enhance the accuracy of the network and improve the quality of identified disease-specific biomarkers 38,108. Chuang *et al*. 5 integrated the gene expression and PPI network data sets to identify biomarkers associated with breast cancer metastasis. The gene expression profiles of two cohorts of breast cancer patients were obtained from literatures, assigned as either ‘metastatic’ or ‘non-metastatic’, and a corresponding human PPI network was constructed by integrating data sets from Y2H, orthology and literature curation experiments. The expression values of each gene were then mapped onto their corresponding proteins in the network to combine the gene and protein data sets. The discriminative potential of candidate subnetwork was computed based on the mutual information between its activity score and the metastatic/non-metastatic disease status over all patients and the significantly discriminative subnetworks were identified by comparing their discriminative potentials to those of random networks. The results showed that known breast cancer genes such as P53, KRAS, HRAS, HER-2/neu and PIK3CA that do not change their expression profile might still play a central role interconnecting genes in the protein network. The identified subnetworks may be more reproducible than individual gene marker selected by traditional approaches, and be better to define metastatic tumours. Systems-based approaches were also used to identify novel biomarkers and understand related mechanisms in a more comprehensive way by integrating protein network with data types or sources, such as phenome 28,40, microRNA 42 or mRNA 33.

The network biomarkers have also been investigated dynamically. Taylor *et al*. 49 examined the dynamic structure of human protein interaction network by analysing ‘intermodular’ or ‘intramodular’ hub proteins that are co-expressed with their interacting partners in a tissue-restricted manner or in all or most tissues. Substantial differences in biochemical structure were observed between the two types of hubs. Hub proteins that displayed dynamic modularity were useful indicators for predicting the outcome of patients with breast cancer. Similar observations were noticed by other groups in the yeast 47,48. Lin *et al*. 119 proposed a network-based approach that integrates PPIs with gene expression profiles and biological function annotations to analyse the interaction networks in different biological states. They found that hub proteins in condition-specific co-expressed PPI networks tended to be differentially expressed between biological states. Applying this method to a cohort of heart failure patients, they identified two functional modules that significantly emerged from the interaction networks that can provide new insights into the cause of dilated cardiomyopathy and might be used as potential drug targets. The dynamic features of network biomarker were also investigated in ageing 98, liver cancer 124, breast cancer 107, glioma 105 and influenza 125.

Table3 summarized some representative network biomarker studies in humans. While the results seemed promising, these studies are method and algorithms oriented; apparently, there is a great need to validate these approaches according to clinical application.

**Table 3:** Examples of network biomarkers studies in humans

Disease	Seeds	PPI data sources	Ref.	Key findings
Breast cancer	Microarray data of two cohorts of breast cancer patients from literature	Database integration of Y2H, prediction and literature curation, includes 11203 proteins and 57235 interactions	5	The identified subnetwork biomarkers increased the reproducibility and accuracy in differentiating metastatic from non-metastatic breast tumours, compared to traditional molecular biomarkers
	Microarray data of two cohorts of breast cancer patients from literature	Database integration of HPRD database and IPA, includes 584 genes and 2280 interactions	35	The identified network biomarkers are highly enriched in biological pathways associated with cancer progression and the prediction performance is much improved when tested across different data sets
Colorectal cancer	Two sets of proteomic targets of colorectal cancer obtained from tissue biopsies	HPRD database includes 9299 proteins and 35023 interactions	33	Integration of complementary data sources can enhance the discovery of candidate subnetworks in cancer that are well-suited for mechanistic validation in disease
	Microarray data of two cohorts of colorectal cancer patients from literature	HPRD database includes 9299 proteins and 35023 interactions	100	The identified subnetwork biomarkers outperformed other biomarkers in predicting metastasis of colorectal cancer and offered insights in the mechanisms of metastasis in cancer
	67 proteins identified from tumour tissue of a cohort of colorectal cancer patients	MetaCorefrom GeneGo Inc. (version 4.6 build 12332)	30	The identified protein subnetwork biomarkers can discriminate late stage cancer *versus* control. Proteins in the subnetworks were associated with the tumour progression or identification
Prostate cancer	A prostate gene data set from literature	Database integration of DIP and HPRD, includes 6509 proteins and 23157 interactions	97	The proposed approach can discover condition relevant functional modules efficiently
Gastric cancer	272 differentially expressed genes in the metastatic gastric cancer	UniHI database	115	The identified subnetwork biomarkers are promising diagnostic markers for liver metastasis of gastric cancer
	Gene expression data from GEO database (ID: GSE27342)	HPRD database includes 9465 proteins and 37039 interactions	111	Identified network biomarkers include 34 genes shown to be directly connected by the gastric cancer-related genes to all phases, and a functional transition from normal phenotypes to cancer phases was demonstrated
Lung cancer	Microarray data from GEO database (ID: GSE4115)	Database integration of BioGRID and HPRD	37	Identified 40 proteins related to lung carcinogenesis. The network-based biomarker was effective in diagnosing smokers with signs of lung cancer
Cardiovascular disease	Mass spectrometry data from major adverse cardiac events patients	HPRD includes 18796 proteins and 37056 interactions	6	The identified network biomarkers can classify the patients with major adverse cardiac events more accurately than traditional molecular biomarkers
	105 heart failure associated proteins from the literature	Database integration of HPRD, BioGRID and MINT	32	The identified network biomarkers support accurate prediction of heart failure and provide novel clue to the underlying mechanisms
	Known inflammation biomarkers from clinical practice and literature	Database integration of DIP, IntAct and MINT	34	Identified a panel of gene biomarkers with high discriminatory capability predicts clinical outcome after myocardial infarction
	CHD microarray data from GEO database (ID: GSE26125 & GSE14790)	Database integration of HPRD, BIND, BioGrid, IntAct and MINT, includes 4761 proteins and 18084 interactions	109	Identified 12 dysfunctional modules from the constructed CHD subnetwork, which provide clue to molecular mechanisms of CHD
	Dilated cardiomyopathy microarray data from GEO database (ID: GSE3586)	HPRD includes 9059 proteins and 34869 interactions	119	Identified two functional modules from the interaction networks. The dynamics of these modules between normal and disease states suggested a potential molecular model of dilated cardiomyopathy
Acute myeloid leukaemia	Microarray data from GEO database (ID: GSE425)	Database integration of HPRD and OPHID, includes 9142 proteins and 41456 interactions	39	Identified AML causing genes most of which were not detectable with gene expression analysis alone because of the minor changes in mRNA level
Asthma	Asthma-associated genes from OMIM database	HPRD	121	The identified subnetworks were consistent with known asthma pathways. Novel asthma associated genes were also identified
Glioma	Microarray data from GEO database (ID: GDS1815)	I2D database, includes 681404 interaction	117	Network biomarkers related to glioma prognosis were identified. MYC expression is positively correlated with lifetime extension
Acute aortic dissection	2737 genes differentially expressed between acute Stanford type A aortic dissection patients and controls	Curated human PPI network, includes 6437 proteins and 258954 interactions	123	Eight PPI hotspots associated with aortic dissection were identified. In particular, JAK2 may play a key role in the occurrence of acute aortic dissection

The table displays biomarkers studies in humans with respective network approach description. In addition, the literature reference for the resource is given.

GEO: Gene Expression Omnibus; OMIM: Online Mendelian Inheritance in Man database; IPA: Ingenuity Pathway Analysis; MetaCore: Data-mining and pathway analysis (http://thomsonreuters.com/metacore/); CHD: Congenital heart disease; OPHID: Online Predicted Human Interaction Database; I2D: Interologous Interaction Database; MYC: myelocytomatosis oncogene.

### Better understanding of molecular mechanism of diseases

The integration of disease-specific molecules into the knowledge-based protein networks and subnetworks is a new and better way to understand mechanistic hypotheses about the causes of disease. The interactions within such subnetworks are often suggestive of functional signalling cascades, metabolic pathways or molecular complexes responsible for or/and contributing to the phenotypes and dysfunction of the disease. Thus, the network approach offers a potent means of mapping the molecular mechanisms underlying complex pathobiological processes. While the networks of genes and proteins present the links and association between them, such knowledge-integrated interaction is relatively defined and fixed. Rather than only the expression, the strength of interactions between genes or proteins varies during the development of diseases. Moreover, DNB is proved to be the leading network to initiate the critical transition during disease progression, and is highly related to causal factors of the disease 8. In this regard, DNBs, by integrating of condition-specific information of network biomarkers at different stages and time-points, promise an improved understanding of the causes of human disease 8,49,119.

Xue *et al*. 98 examined the modular structure of the protein interaction networks during the ageing of fruitfly and human brains and found two modules associated with the cellular proliferation to differentiation temporal switch that display opposite ageing-related changes in expression. This particular study provides a modularized network view of the ageing process and found the dynamic network stability might be associated with the ageing. Such a dynamic network view provides a molecular explanation for the stochastic nature of ageing, that is, isogenic population age at vastly different paces, for the states of the network can be differentially affected by developmental and environmental factors. Li *et al*. 126 constructed dynamic physical and functional protein interactions network regulating the production of type I interferon (IFN) and identified 22 unique genes that regulated NF-kB and ISRE reporter activity, viral replication or virus-induced IFN production. Among them, mind bomb (MIB) E3 ligases played a role in K63-linked ubiquitination of TBK1kinase that phosphorylates IRF transcription factors controlling IFN production. MIB genes were found selectively controlled responses to cytosolic RNA viruses, and MIB deficiency reduced antiviral activity. This study established the role of MIB proteins as positive regulators of antiviral responses and demonstrated that mapping a dynamic physical and regulatory network of type I IFN can be a valuable source for understanding the connections between innate immunity and other cell processes.

Based on the computational algorithm (composite index *I*) described above, researchers found two dynamical network biomarkers which can be separately used to predict the peri-insulitis of the early stage of disease and the onset of type 1 diabetes with overt hyperglycaemia 127. These two DNBs were adopted to analyse and revealed that mitochondrion electron transport induces the apoptosis function of the second DNB and pushes the peri-insulitis to diabetes. Li *et al*. 128 identified tissue-specific DNBs corresponding to the critical transitions occurring in liver, adipose and muscle during type 2 diabetes mellitus (T2DM) progression, and found two different critical states during T2DM development, characterized as responses to insulin resistance and serious inflammation respectively. The identified DNB genes are significantly associated with T2DM, either to be the disease genes or participate in important biological processes related to the T2DM development, such as response to insulin stimuli, abnormal lipid metabolism and immune system response. DNB genes were also found tend to be located at the upstream of pathway rather than the consequence so that DNB genes act as the causal factors to drive the downstream molecules to change their transcriptional activities. These studies demonstrated that DNB approaches can detect the early warning signals for detecting the normal and pre-disease states, and provide insights to the molecular mechanism of disease phenotype or complex physiological processes.

Fang *et al*. investigated the relationship of cigarette smoking and lung cancer development 44. The disease states (tumour or normal), smoking states (current smokers or non-smokers or former smokers), and the disease stage (stages I–IV) were pair-wise compared using a novel strategy that incorporates network-based approach with gene set enrichment analysis. They identified panels of gene candidates that involve in cell proliferation and drug metabolism, such as cytochrome P450 and WW domain containing transcription regulator 1, in smoking or lung cancer development. Pathways of cell cycle, DNA replication, RNA transport, protein processing in endoplasmic reticulum, vascular smooth muscle contraction and endocytosis were found commonly involved in smoking and lung cancer. Furthermore, semaphorin 5A and protein phosphatase 1F were identified as the common genes represented in major hubs in both the smoking and cancer-related network. This study provides an excellent example not only to understand the complex and dynamic relationships between cigarette smoking and lung cancer but also to reveal molecular mechanisms of cancer initiation and progression at a network level.

## Correlation between network biomarkers and clinical informatics

Network approaches allow an accurate identification of biomarkers. DNBs have the advantage to demonstrate pathophysiological changes at different stages and periods. One of the most challenges is to translate network biomarkers into clinical application and validate the disease specificity 9,10. The biological measurements need to be statistically and mechanistically correlated with clinical information. But conventional clinical measurements, such as complaints, history, symptoms and signs, physical examinations, laboratory tests, medical imaging and therapies, are descriptive and rarely integrated, comparing with biological data. As such, clinical bioinformatics was proposed to combine clinical phenotypes with human tissue-generated bioinformatics, to understand molecular mechanisms of the disease, and to define relationships between collectively direct global function with clinical measurements 9. Defined as ‘the clinical application of bioinformatics-associated sciences and technologies to understand molecular mechanisms and potential therapies for human diseases’, clinical bioinformatics emphasizes the association and specificity complex biomedical data sets with the disease complexity of patient information. It suggests that the integration of biology data with clinical informatics can be a new way to validate and optimize disease-special network biomarkers 10. It would be even more values if clinical bioinformatics can integrate network-based approaches to prioritize disease-specific interaction subnetworks between gene–gene, gene–protein, or protein–protein with disease signature and clinical phenotypes, to improve the accuracy of clinical diagnostics and prediction.

The strategies to integrate biological and clinical data have been proposed and are still under the rapid development 129,130. Using a clinical bioinformatics approach, Schwarz *et al*. 131 quantified relationships between specific variables of patients with schizophrenia (*i.e*. cerebrospinal fluid and serum samples, obtained from two different profiling platforms and standard laboratory tests) as networks, and detected a subgroup of patients featuring remarkable abnormalities in a network of serum primary fatty acid amides. The identified disease-associated patterns of biomarkers were suggested to be able to describe the complicity of psychiatric diseases. This particular study demonstrated that simultaneous evaluation of clinical data and molecular biology data *via* a clinical bioinformatics approach could improve the understanding of complex diseases and lead to better diagnosis, prediction and therapy.

Because of the large and independent nature of the clinical data, the application of controlled vocabulary and ontology for the standard nomenclature of clinical trial data is critical and important for clinical data integration 129. In a preliminary study, Chen *et al*. 22 utilized chemokine multiplex antibody array to detect inflammatory mediators in the circulation of patients with acute exacerbation (AECOPD) or stable condition (sCOPD) of chronic obstructive pulmonary disease to correlate DNBs with clinical informatics. Clinical informatics, which translates clinical descriptive information into the digital data, was achieved by a digital evaluation score system (DESS) for assessing severity of the patients. DESS is a score index established by senior chest physicians that take into account patient symptoms, signs, doctor examination, clinical imaging and biochemical analyses in patients with AECOPD or sCOPD. For the assessment of the severity, each component was assigned with different scores as 0, 1, 2 and 4. The score of 4 (maximal value) indicates far more above normal range or more severe condition, while 0 (minimal value) means the variable is within physiological range. The value of 3 was missed in the scoring system for exponential values to better define the severity stages. The points of each variable were added after compiling patients' data and DESS values ranged from 0 to 264, of which higher scores indicate severer conditions. By integrating proteomics-based bioinformatics with clinical informatics, disease-specific biomarkers in the circulation were scanned and a multi-scale predictive model was established. The authors identified 13 mediators (BTC, IL-9, IL-18Bpa, CCL22, CCL23, CCL25, CCL28, CTACK, LIGHT, MSPa, MCP-3, MCP-4 and OPN) that could discriminate AECOPD patients from both healthy and sCOPD patients.

**Fig 4:**
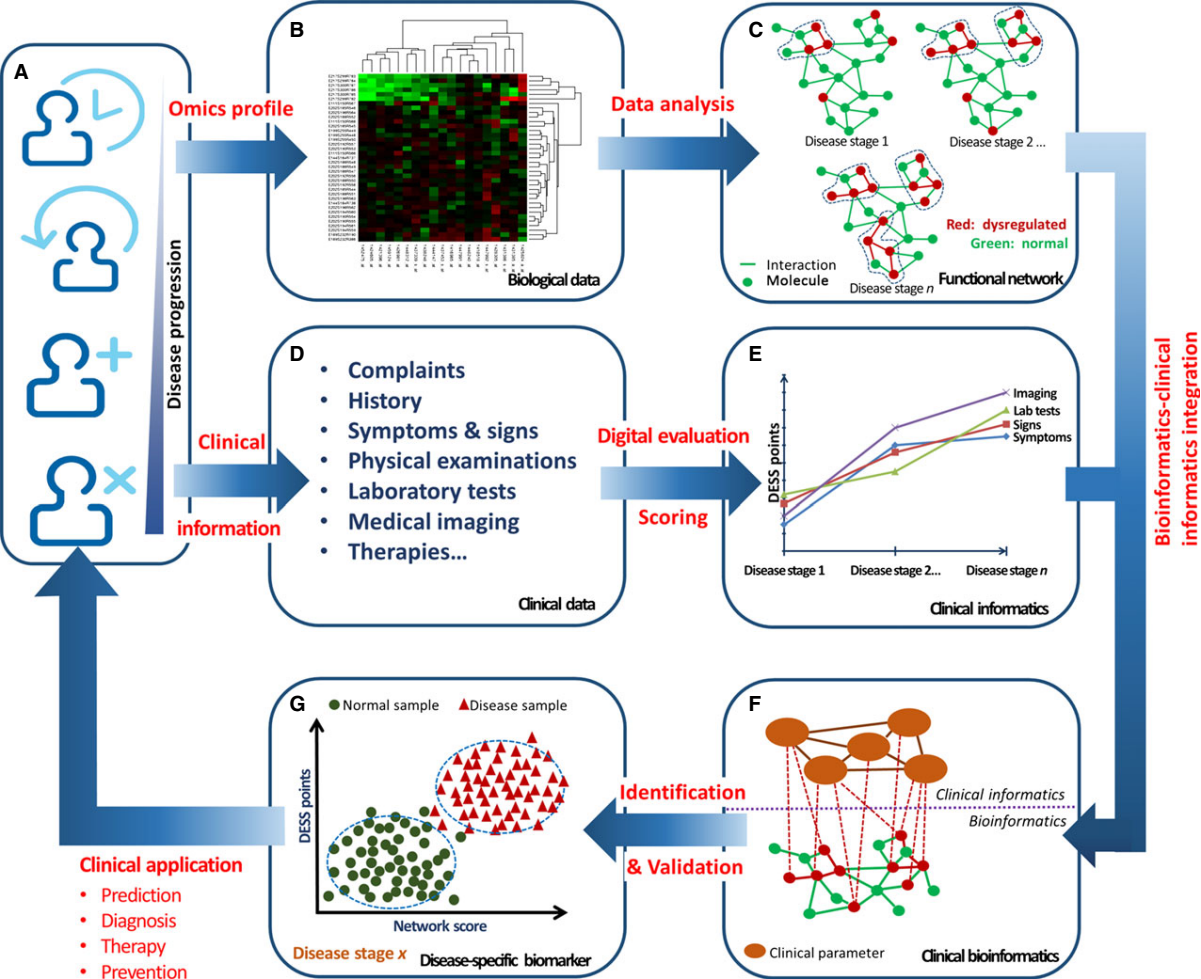
A proposed workflow of disease-specific biomarker identification by integration of bioinformatics and clinical informatics. Both the global expression profiles (B; genomics, proteomics, *etc*.) and the clinical data (D) of a disease phenotype at different stages (A) are obtained. Disease-associated functional networks (C) are measured by bioinformatics, while clinical informatics (E) is generated through a digital evaluation score system (DESS). By integrating bioinformatics and clinical informatics, the molecular-phenotype networks are measured using different methods to score, rank and identify candidate biomarkers (F). The identified disease-specific biomarkers are then validated to differentiate a disease phenotype from a normal phenotype (G) for clinical application to develop predictive, diagnostics and preventive methods for personalized medicine.

Using similar approach, the authors further investigated the potential correlation of proteomic profile with clinical informatics in COPD patients. Plasma samples from 18 patients including healthy individuals or patients with sCOPD or AECOPD were collected to measure 507 inflammatory mediators using antibody microarray 24. Clinical descriptive information was translated into digital data by DESS for severity assessment. Twenty mediators were significantly different between three groups, of which Cerberus 1, inhibin B, osteoactivin and thrompoietin were firstly reported in COPD and AECOPD. Ten cytokines such as Cerberus 1, Growth Hormone R, IL-1F6, IL-17B R, IL-17D, IL-19, Lymphotoxin beta, MMP-10, Thrombopoietin and TLR4 were found inversely correlated with DESS scores. A down-regulation of systemic inflammatory responses was indicated in the occurrence of AECOPD. These studies demonstrated that the integration of omic profiles with clinical informatics as part of clinical bioinformatics is important to discover, validate and optimize disease-specific and disease-staged biomarkers. The proposed protocol for disease-specific biomarker discovery by integrating bioinformatics and clinical informatics is illustrated in Figure4. Firstly, the expression profiles and the clinical data of a disease phenotype during disease precession are obtained. Secondly, the disease-associated networks are measured by bioinformatics, and clinical informatics is generated through a digital evaluation score system. Thirdly, the molecular-phenotype networks are then measured and ranked by integration of bioinformatics and clinical informatics to identify candidate biomarkers. And lastly, the identified disease-specific biomarkers are validated for clinical applications.

## The advantages and constraints for clinical application

Protein-based network biomarkers with systems information can provide a more precise and complete profile of cellular changes in human diseases. As proteins perform the major cellular functions essential to signal transduction that role cell growth, differentiation, proliferation and death, protein-based network biomarkers are critical in providing valuable information at the post-translational level that can be used to establish diagnosis or prognosis of a disease and to develop personalized therapeutics, with favourable clinical feasibilities. The effectiveness of protein-based network biomarkers has been demonstrated in the context of various diseases.

On the other hand, a number of proteomics-associated challenges should be bear in mind. For instance, proteomics experiments typically screen only a limited fraction of proteins, in particular, gel-based expression experiments are most likely to detect high abundance proteins. The human PPI data are still incomplete and variable because of different curated collections. Therefore, the advantages of the network biomarker discovery include not only easily avoiding data noises by knowledge-based network but also deriving high confident network biomarkers.

A number of challenges still exist in every step of the network biomarker development pipelines, despite of technological advances. Protein post-translational modification and alterations in protein stability may influence network modularity on a global scale during disease progression. Most high-throughput methods can suffer from high false-positive or -negative rates that may lead to misclassifications. Large-scale networks are not specific to diseases or clinical phenotypes. The level of certainty is constrained by the issues of data collection, interpretation of large size of the proteome, and the diversity of cells and tissues. On the other hand, noise generated during different network methodologies integration remains a major constraint to correctly interpret complex networks and needs to be critically evaluated and managed. Thus, the principles used in network discovery, validation and development remains to be further defined and quantified, and the development of novel and reliable statistical tools for the network environment is urgently needed. Also, the lack of standardized vocabularies for a definitive translation of networks into the clinical arena represents a main challenge in the integration and interpretation of clinical bioinformatics. Another important challenge is to translate DNBs into the understanding of clinical phenotypes, molecular mechanisms of disease development and progress, and development of therapeutic strategy 10.

Despite these limitations related to knowledge incompleteness and uncertainty in the network inference process, the characterization of complex biological phenomena on the basis of functional modular architectures and topological parameters present us with new opportunities to improve our understanding of the aetiology, evolution and therapeutics of the diseases. To reach clinical application, the advantages and disadvantages of protein-based network biomarkers should be furthermore investigated to evaluate the potential values of network biomarkers in the development. We believe that clinical bioinformatics can play an important role in identification and validation of disease-specific DNBs.

## Prospective and conclusions

Better biomarkers are urgently needed to disease detection, diagnosis and prognosis. Network approaches have revolutionized the traditional ways of biomarkers discovery and offered a powerful way for pathway mapping and development of disease-specific biomarkers. Although challenges exist in steps of the network biomarker development, network biomarkers are proving to play importance roles in disease-causing genes prediction, disease-related subnetworks identification, disease classification, disease *in vitro*/*in silico* modelling, drug discovery and target screening, and ultimately improving the outcome and life quality of patients.

In the era of network medicine, new biomarker discovery depends on a comprehensive view of transcriptome, genome, proteome, and metabolome, or diseaseome. The dynamic nature of human protein interaction network because of the diversity and regulative structure of post-translational modifications, gives in-depth insight into disease mechanism. Scientists and physicians are facing more challenges to keep the pace with the growing availability of a variety of high-throughput data. Global efforts are been done to improve our understanding of diseases through integrative approaches to translate science into the clinical practice 132–134. With the development of clinical bioinformatics, biomarker discovery should not only integrate different types of omics data, but also consolidate of such molecular biological measurements with clinical phenotypes. The development of profiling technologies, biological databases, data mining, biostatistics and clinical bioinformatics will tremendously speed up the identification, validation and development of disease-specific network biomarkers and DNBs.
